# Proteomic anaysis of aged microglia: shifts in transcription, bioenergetics, and nutrient response

**DOI:** 10.1186/s12974-017-0840-7

**Published:** 2017-05-03

**Authors:** Antwoine Flowers, Harris Bell-Temin, Ahmad Jalloh, Stanley M. Stevens, Paula C. Bickford

**Affiliations:** 1 Department of Neurosurgery and Brain Repair, USF Health Morsani College of Medicine, University of South Florida, 12901 Bruce B Downs Blvd., Campus Box MDC-78, Tampa, FL 33570 USA; 2 Department of Molecular Pharmacology and Physiology, USF Health Morsani College of Medicine, University of South Florida, Tampa, FL USA; 3 Department of Cell Biology, Microbiology, and Molecular Biology, University of South Florida, Tampa, FL USA; 4 Research Service, James A Haley VA Hospital, Tampa, FL USA

## Abstract

**Background:**

Age is the primary risk factor for many diseases. As such, age is a critical co-factor for examination in order to understand the progression and potential intervention in disease progression. Studies examining both the phenotype and transcriptome of aged microglia demonstrated a propensity for the development of a pro-inflammatory phenotype. Less well studied is the concomitant blunting of anti-inflammatory aspects of microglial function with age which also impact plasticity and repair in the CNS.

**Methods:**

This study utilizes mass spectrometry-based proteomics to compare primary microglia from young and aged animals.

**Results:**

This study revealed alterations in three clusters of inter-related proteins. The three pathways were inflammatory signaling, mitochondrial function, and cellular metabolism. Analysis of these clusters identified the protein rapamycin-insensitive companion of mTOR (RICTOR), a component of the mTORC2 complex, as a novel upstream regulator of several biological functions that are altered with age and potentially linked to phenotype development. A decrease in mTORC2-dependent AKT S473 phosphorylation, as assessed by insulin growth factor (IGF) treatment, was observed in aged microglia. This novel finding was confirmed by genetic manipulation of the microglial cell line. BV2 cells with diminished RICTOR displayed a phenotype that was strikingly similar to that of aged microglia. This finding is particularly relevant as the mTOR pathway already has a number of pharmacological modulators used clinically.

**Conclusions:**

The results suggest that microglia from aged mice show changes in cellular metabolism and energy regulation that might underlie the alterations in inflammatory signaling. Modulation of one pathway identified in our bioinformatic analysis, RICTOR, may provide an avenue by which deleterious aspects of the aging microglia can be attenuated. If successful, this could mean potentially delaying or diminishing the progress of diseases for which progressive inflammation is involved.

**Electronic supplementary material:**

The online version of this article (doi:10.1186/s12974-017-0840-7) contains supplementary material, which is available to authorized users.

## Background

Aging is a complex phenomenon that profoundly alters the landscape of our physiology. These alterations play a role in creating an environment in which chronic and degenerative diseases can progress [36]. Age is a leading risk factor for a wide number of diseases including cancer, dementia, and neurodegenerative diseases. Aging is best described as the progressive loss of integrity leading to loss of function in organ and tissue systems. The effects of aging are significantly pronounced in the immune system. In the peripheral immune system, there is a loss of naïve T cells with age, which accompanies a dysregulated cross talk between T cells and B cells in the lymph nodes [29]. The inflammatory response is a well-orchestrated set of events that coordinate multiple stages of cellular differentiation, movement, and activation. There is an apparent loss of this coordination in aged animals, culminating in impaired cross talk between T cells and macrophages [35, 47]. Aged macrophages exhibit a loss of functional plasticity and appear to be primed towards a pro-inflammatory phenotype [41]. In addition, they have an altered transcriptional profile in response to receptor activation, likely contributing to their loss of plasticity [35]. In the CNS, the immune landscape differs from that of the periphery. While the CNS is no longer believed to be as “immune privileged” as it once was, the cells that make up the majority of the immune landscape are the resident macrophage, the microglia. These cells are critical throughout life, including during development for proper CNS function. In addition to their roles as immune cells, they support synaptic function through pruning, a necessary process for learning consolidation [9]. Microglia are regulators of neurogenesis and provide vital support roles to neurons through their production of neurotrophins during periods of stress or injury [48].

Microglia, like immune cells in the periphery, undergo dramatic changes with age [11]. The aged CNS is characterized by higher levels of inflammatory cytokines and chemokines even in the absence of insult [11]. Aged microglia have dystrophic morphologies and loss of dendritic processes that are vital for communication with their environment and the maintenance of synapses [13]. There is evidence that these changes contribute to the cognitive decline normally seen with age, which in severe cases can result in cognitive impairment and dementia [33]. There is also evidence that this loss in microglial function is a necessary step for the development of neurodegenerative disease [10]. Aged microglia exhibit exaggerated and prolonged responses to inflammatory stimuli and are resistant to regulation by anti-inflammatory stimuli such as IL-4 [15, 19]. This blunted response to anti-inflammatory stimuli may play a role in the reduced ability to mount repair mechanisms and is underexplored in the aging field.

Studies of aged microglia at the system level have revealed altered transcriptional patterns that further confirm a pro-inflammatory phenotype [23] and a reduced response to various signals. The majority of studies so far have analyzed the transcriptome using a variety of tools including RNA sequencing and DNA microarrays; however, to the best of our knowledge, there has been no detailed, global-scale analysis to determine age-related effects at the proteome level.

In this study, the effects of age on the microglial proteome was examined through mass spectrometry-based proteomics using a modified stable isotope labeling by amino acids in cell culture (SILAC) approach. Protein expression networks in pure populations of microglia from young and aged mice were examined using functional clustering analysis. From these clusters, cellular systems contributing to the underlying changes in biology were determined. Next, proteins were enriched for canonical pathways in order to identify signaling pathways with the greatest impact on the age-related microglial phenotype. Upstream regulator analysis was performed to identify molecules whose activity could result in the downstream expression changes of proteins enriched in the identified pathways. From these potential regulators, the mTORC2 component protein, rapamycin-insensitive companion of mTOR (RICTOR), was identified as a promising target for regulation of the age-dependent dysfunction of microglia. Our proteomic dataset represents the first comprehensive age-related microglial protein expression profile and, in conjunction with bioinformatic analysis, has provided molecular insight into phenotypic changes that occur in microglia upon aging.

## Methods

### Cell culture and treatments

Primary microglia were grown in base media of DMEM supplemented with F12, 10% heat inactivated fetal bovine serum, glutamine (glutamax 2 mM), 100 μg/ml penicillin, and 100 U/ml streptomycin. Cells were allowed 3 days in culture at which time dendritic arbors representative of a resting phenotype were easily discernible. For phoshorylation studies, cells were cultured overnight in DMEM as described above with only 1% FBS. After serum starvation cells were treated with 100 ng/ml IGF in serum-free media for 15 min prior to lysis with RIPA buffer supplemented with calyculin A and protease inhibitors. For western blots assessing phenotypes, cells were cultured overnight in 1% FBS DMEM. Cells were subsequently treated with 20 ng/ml TNFα and IL-4 (Sigma) for 24 h.

### Primary microglia extraction

Primary microglia were obtained from young (3–5 months old) and old (20–24 months old) animals. Mice were euthanized with CO_2_ according to IACUC standard protocol. A single-cell suspension was obtained using Miltenyi Biotec’s neural tissue disassociation kit (P) (130-093-231). Briefly, brains were removed and placed in cold HBSS w/o Ca++ and Mg+ on ice. Using a scalpel, the brains were mechanically disassociated in a petri dish and then transferred to a 15-ml tube and spun at 200*g* for 2 min. The tissue was enzymatically digested according to manufacturer’s protocol to obtain a single-cell suspension. Primary microglia were isolated using Miltenyi Biotec’s LS magnetic columns and CD11b magnetic beads (130042401 and 130093634, respectively). This procedure yielded between 200K and 300K cells with 95% purity as confirmed by ICC.

### Super-SILAC processing and mass spectrometric analysis

Prior to lysis, cells were washed with PBS and collected with a rubber cell scraper. Cells were then lysed in 4% SDS in 100 mM Tris-HCl, pH 7.6, and 100 mM dithiothreitol at 95 °C for 4 min. The Super-SILAC BV-2 internal standard was prepared by culturing immortalized mouse microglia in SILAC DMEM supplemented with 10% SILAC dialyzed FBS, 1 ml of 100× PSG, and 100 mg/L each of heavy ^13^C_6_ lysine and ^13^C_6_, ^15^N_4_ arginine. Seven doublings were allowed to achieve proper incorporation before 24 h in serum-free media. Separate flasks were then treated with 30 ng/ml LPS, 30 ng/ml IL-4 and 10 ng/ml IL-13, 10 ng/ml IL-10, or 50 mM ethanol for 24 h, in addition to untreated cells. Cells were lysed in 4% SDS, 100 mM Tris-HCl, pH 7.6, and 100 mM dithiothreitol for 5 min at 95 °C prior to probe sonication and clearance of lysate via 15,000*g* microcentrifugation for 5 min. Protein assay was performed via 660 nM protein assay with ionic detergent compatibility reagent and lysates combined together in equal protein amount to create the internal standard. Microglia isolated from young and old mice were processed using the same approach. Equal quantities of the stable isotope-labeled BV2 internal standard were added to the microglial cell lysates and digested via the FASP procedure[18]. Briefly, cells were exchanged into 9 M urea and alkylated with 10 mM iodacetamide in a 30-kDA Microcon Forensic Column (Millipore) across multiple 14,000×*g* centrifugations prior to exchange into 25 mM ammonium bicarbonate. Proteins were digested overnight using a 1:100 ratio of Mass Spectrometry Grade, TPCK-treated trypsin (Promega) prior to collection into a new tube. Samples were desalted on C18 SPE columns, concentrated in a vacuum concentrator, and resuspended in 0.1% formic acid prior to mass spectrometric analysis.

Microglia digests were separated on an Acclaim PepMap C18 (75 μm × 50 cm) UPLC column (Thermo) using an EASY-nLC 1000 with a gradient time of 120 min (2–40% acetonitrile in 0.1% formic acid). Mass spectrometric analysis was carried out on a hybrid quadrupole-Orbitrap instrument (Q Exactive Plus, Thermo) using data-dependent acquisition in which the top ten most intense ions were selected for MS/MS. Full scan and MS/MS resolution was 70,000 and 17,500, respectively.

### Statistical, pathway, and upstream regulator analysis

High-resolution mass spectrometric data were analyzed on the MaxQuant processing suite, version 1.5.4.1. Spectra were searched against the Uniprot reference database for *Mus musculus* using the MaxQuant built-in peptide identification algorithm, Andromeda. Trypsin was specified as the digestion protease with the possibility of two missed cleavages. Acetylation (protein N-terminus) and oxidation of methionine were set as default variable modifications while carbamidomethylation of cysteine residues was set as a fixed modification. Additional modifications included the SILAC labels used for the spike-in standard. Other database search parameters included trypsin/P as the enzyme used with the possibility of two missed cleavages. Also, 20 ppm (first search)/4.5 ppm (recalibrated second search) mass tolerance for precursor ions and 20 ppm mass tolerance for fragment ions was specified in the database search. Intensities for all peptides were assigned by MaxQuant using full scan mass spectra, and ratios between heavy and light SILAC partners were calculated. Identifications were filtered using a target/decoy strategy employing reversed sequences with a false discovery rate of 1% for peptides and proteins. The MaxQuant ProteinGroups file was then analyzed using the Perseus (version 1.5.4.1) processing suite. The protein list was log2-transformed and filtered to include proteins that contain 50% valid values (i.e., detectable ratio). For proteins that did not generate ratio values or were outside a range of consistent ratio detection (i.e., not enough replicates to perform *t* test), presumably due to proteome differences between primary microglia and BV2 cells, the raw intensity values for the “light” peptides were used to perform label-free quantitation of relative protein expression. In the label-free approach, missing intensity values for lower abundance proteins were replaced with the imputation function in Perseus using default parameters. Final ratios represent old/young for each protein (mean, *n* = 5 for old and *n* = 5 for young) where SILAC-generated ratios were calculated through a ratio-of-ratio calculation and label-free ratios were calculated from raw intensity values. MaxQuant results were also input into the program Scaffold (version 4.6.2) for final filtering of protein identification results and visualization of mass spectrometric data. In both the SILAC and label-free approaches, statistical significance was established at *p* < 0.05 using the Student’s *t* test on log2-transformed ratios or intensity values. Proteins that were identified as significant (no FDR correction was applied in order to improve depth of coverage and enhance bioinformatic output) were entered into Ingenuity Pathway Analysis suite to determine localization, molecular function, and protein interaction pathways. Upstream regulator analysis identifies upstream transcriptional regulators that can explain the observed gene expression changes in a user’s data set. For each potential regulator, two statistical measures are taken, an overlap *p* value and activation *z*-score. The *p* value calculates the overlap between a known regulator and dataset targets of that gene or protein through a Fisher exact test while the *z*-score infers likely activation states based on a comparison model [30] where *z* > 2 or <2 indicates activation or inhibition, respectively. Further filtering of the dataset to control for type I error was performed using Welch’s *t* test on log2-transformed ratios (SILAC) or intensity values (label-free) in addition to implementation of a *z*-score cutoff. The *z*-score was calculated as [(*t* test difference of individual protein) − (median *t* test difference of dataset)] / (standard deviation of *t* test difference of dataset).The criteria of |*z*-score| >1 and *p* < 0.05 with Welch’s *t* test were utilized as a second more stringent filtering approach.

### RT-PCR

After treatment as described above, cells were lysed and RNA was collected using the RN-easy plus kit (QIAGEN). Concentration and quality were determined using a nanodrop analyzer (thermo). One hundred nanograms of RNA was mixed with vilo superscript cDNA reverse transcription mix (Thermo) to create a cDNA template. RT-PCR was performed using the power up SYBR Green mastermix (Applied Biosystems) on a step-one plus real-time PCR machine (Applied Biosystems). The following “fast” cycling conditions were used: 2 min 50° C, 1 min 95 °C, 15 s 95 °C, and 3 s 60 °C with read. This was repeated for 40 cycles followed by a melt curve. The primers used were TNFα, IL1β, IL6, MARCO, ARG1, IGF1, ACTB, and YM1 (IDT Primetime Primers, ref seq NM_013693, NM_008361, NM_031168, NM_010766, NM_007482, NM_1055770, NM_009892) at a concentration of 500 nM.

### Western blot

The primary cell treatments described above were performed and the cells collected and lysed. Protein concentrations were determined by BCA assay. Western blots were run using the Wes from Protein Simple. This microfluidic-based assay is a highly quantitative method for determining protein levels from dilute lysates. Cell lysates were diluted to 20 μg/ml, and a size assay was run on a 25-well plate. The assay parameters were as follows: 25 min separation time, 375 V, 5 min antibody diluent, 60 min primary antibody incubation, and 30 min secondary incubation. The following antibodies were used: 1:50 AKT (rabbit, Cell Signal), 1:50 pAKT (s473) (rabbit, Cell Signaling), and GAPDH 1:2000 (rabbit, Cell Signaling). Sample intensity was calculated using the compass software, and quantity was determined using the area under the curve calculation.

## Results and discussion

### Proteomics-based identification of aging-related proteins

**Fig. 1:**
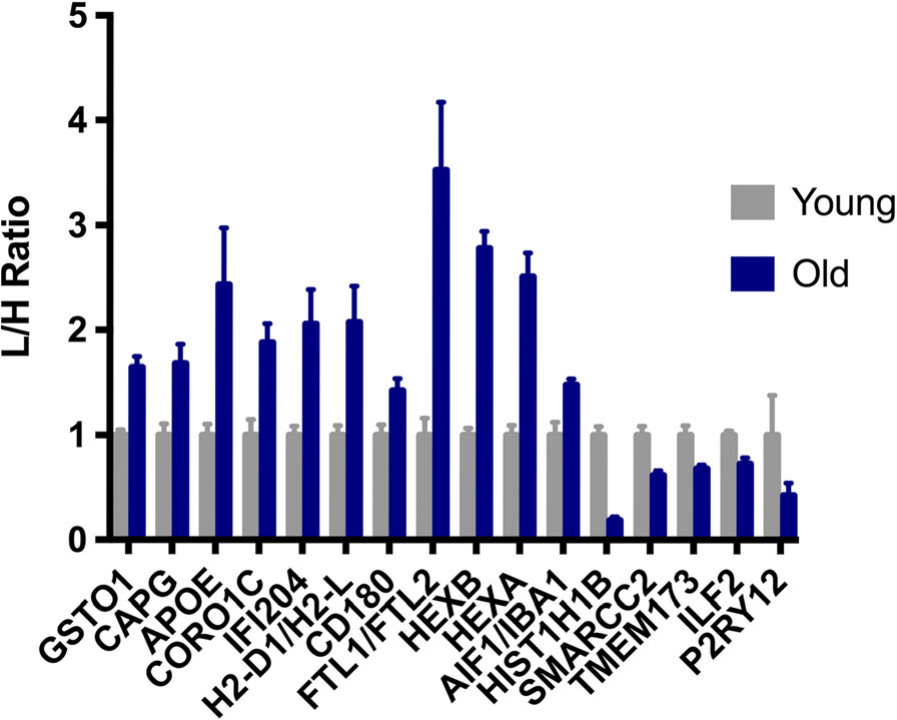
Age-related differential expression of selected microglial proteins. SILAC or label-free ratios were determined by MaxQuant and then normalized to the young group for each individual protein. *Error bars* represent SEM and statistical significance was determined by Welch’s *t* test (*p* < 0.05) and |*z*-score| >1

SILAC is a metabolic labeling approach that allows for relative protein quantitation with fairly high accuracy. However, this method typically requires proliferating cells in order for the heavy-labeled amino acids to integrate into the proteome. We have adapted this method to allow for quantitative proteomic analysis of primary microglia in which heavy-labeled protein from the microglial BV2 cell line was utilized as a spike-in standard. This approach allowed us to overcome the challenge of integrating heavy-labeled amino acids into primary microglia isolated from adult mice in which incorporation efficiency is limited by post-isolation culture time. To overcome differences in coverage between the BV2 proteome and primary cells (outside of range for SILAC-based quantitation), we performed label-free quantitation using intensities measured for primary microglia. After input of database search results into the Scaffold program, our mass spectrometric analysis identified 1944 proteins in 1823 clusters without consideration of modifications (e.g., post-translational or SILAC) in order to establish proteome depth of coverage for the primary microglia. Criteria for protein identification included a threshold of <1% FDR at the protein and peptide level with a minimum of two unique peptides identified for each protein. When considering modifications, which includes SILAC labels for the BV2 proteome, 2843 proteins (57 of which are potential contaminants) in 2713 clusters were identified. To determine differentially expressed proteins, we performed Student’s *t* test between the young and aged groups. The proteins at this significance threshold (271 total differentially expressed proteins, Additional file 1: Table S1) were utilized for subsequent bioinformatic analysis. No multiple testing correction was applied in order to maximize proteome depth of coverage typically necessary to enhance bioinformatic output. However, we have provided a separate table (Additional file 2: Table S2) that filters differentially expressed proteins by both Welch’s *t* test as well as *z*-score (see the “Methods” section). This type of filtering approach has been shown to be less stringent than multiple testing correction methods (e.g., Bonferroni or Benjamini-Hochberg) while still maintaining adequate FDR and sensitivity [43]. Several differentially expressed proteins (out of 156 total differentially expressed proteins from the second filtering approach) related to microglial function are shown in Fig. 1. These include those that are known microglial markers (e.g., AIF1/IBA-1 and HEXB) and/or involved in immune response (e.g., H-2 class I histocompatibility antigen and interferon-related proteins). IBA-1 is a component of the MHC class III complex and is upregulated in the brain by microglia during an inflammatory response. Previous studies of the aged hippocampus observed higher number of microglia expressing MHC molecules, including IBA-1, suggesting a pro-inflammatory state [13]. The overall direction of fold change based on some of these selected proteins suggests an amplified activated state and/or pro-inflammatory posture of microglia isolated from old mice when compared to young animals. Other proteins were selected to demonstrate quantitation results for those involved in biological processes implicated in age-related microglial functional changes discussed later. In order to determine molecular pathways and potential regulatory mechanisms associated with age-related microglial priming, we then used the Ingenuity Pathway Analysis platform and DAVID GO database to analyze relationships between cellular pathways and systems.

### Altered functional processes identified in aged microglia

Functional processes were identified using the functional categories in the GO database [2] that were represented by a statistically higher proportion of proteins in the differentially expressed group than would be predicted by chance. To accomplish this, we used the DAVID platform. The functional annotation clustering tool was also used to group overrepresented functional processes based on overlap among populating proteins. Differentially expressed proteins that were up- or downregulated were uploaded and analyzed separately. Table 1 contains overrepresented functional processes for upregulated and downregulated proteins as well as a list of the proteins involved in each. To reduce redundancy, Table 1 only contains processes that are the most informative and characteristic of each cluster. The full list is available in the Additional files 1 and 2.

**Table 1:** Functional processes identified from proteins differentially expressed with age

Upregulated	Downregulated
Oxidative phosphorylation—UQCRC2, NDUFB3, ATP5D, TCIRG1, UQCRC1, COX7A2, NDUFA7, ATP5F1, COX4I1, COX6C, SDHA, ATP6V1C1, ATP6V1A, NDUFS5, COX6B1, ATP6V0D1	Spliceosome—NCBP1, TCERG1, PRPF8, U2AF2, TRA2B, SNRNP200, PRPF3, HNRNPC, DDX5, HNRNPU, SF3B2, SNRPG, PRPF40A
Oxidoreductase—HSD17B11, UQCRC2, HSD17B10, CYB5R1, ME2, GLUD1, PGD, OGDH, PRDX1, FTH1, HADHA, ACOX3, IVD, CAT, COX7A2, ACADS, COX4I1, CBR4, TECR, COX6C, SOD2, SDHA, DHRS1, CYBA, BLVRB	Nuclear lumen—LMNB1, MTA2, PRPF3, DDX5, CBX5, HNRNPL, NONO, SET, TCERG1, HNRNPH2, ILF2, FRG1, PRPF8, ANP32A, RRS1, ACTL6A, RUVBL1, HNRNPH1, RBM14, LBR, PRPF40A, SMARCA4
Mitochondrion—UQCRC2, ATP5D, UQCRC1, COX7A2, ME2, GATM, ACADS, GLUD1, ATP5F1, ECHS1, COX4I1, OGDH, HADHA, HADHB, SOD2, SDHA, ACOT9, IVD, OXCT1, ATPIF1, HMGCL	Helicase activity—DDX17, SNRNP200, RUVBL1, DDX5, CHD4, SMARCA4
Hydrogen ion transmembrane transporter activity—TCIRG1, ATP5D, ATP6V1C1, ATP6V1A, COX7A2, COX6B1, ATP5F1, COX4I1, ATP6V0D1, COX6C	Nucleotide binding—RAB5B, TRA2B, U2AF2, HNRNPL, GPD1L, NONO, HSPH1, DDX17, AGPS, TARDBP, TUBB5, ACTL6A, HNRNPC, CHD4, CSTF2, STK24, ELAVL1, ATP1A1, DDX5, CRYZ, HNRNPR, HNRNPH2, ILF2, MAPK14, PSMC1, SNRNP200, RUVBL1, RBM14, SMC1A, HNRNPH1, SMARCA4, MDH1
Regulation of actin cytoskeleton organization—DBNL, CORO1A, ARPC3, ARPC2, CAPG, MYO1F, ARPC5, CAPZB	Chromatin organization—SET, SMARCD2, HIST1H1B, MTA2, SMARCC2, ACTL6A, RUVBL1, RBM14, CHD4, CBX5, SMARCA4
Lysosome—LAMP1, NPC1, CTSZ, LAMP2, LIPA, HEXA, HEXB, ARL8A, ACP2, CTSB, ARL8B, SCARB2	Regulation of transcription—SUB1, MTA2, TERF2IP, DDX5, CBX5, NONO, FUBP3, TCERG1, SMARCD2, ILF2, HNRNPUL1, TARDBP, MAPK14, SMARCC2, ANP32A, ACTL6A, RUVBL1, RBM14, CHD4, SMARCA4
Generation of precursor metabolites and energy—UQCRC2, NDUFB3, ATP5D, TCIRG1, UQCRC1, NDUFA7, ATP5F1, HK2, OGDH, SOD2, SDHA, ATP6V1C1, ATP6V1A, CYBA, NDUFS5, GOT1, CAT, ATP6V0D1	Nucleocytoplasmic transport—NCBP1, KPNA6, NUTF2, IPO9, KPNA3
Valine, leucine, and isoleucine degradation—HSD17B10, ACADS, IVD, OXCT1, ECHS1, HMGCL, HADHA, HADHB	Proteasome complex—PSMB4, PSMA6, PSMC1
Response to oxidative stress—GATM, APOE, CAT, GCLM, PRDX1, SOD2	DNA repair—NONO, DDB1, SMC1A, APEX1
Immune effector process—PTPRC, CD47, MYO1F, FCER1G, INPP5D, PRDX1	Coenzyme binding—GPD1L, DBT, AGPS, CRYZ, MDH1
Cell chemotaxis—CORO1A, FCER1G, NUP85	Chromatin remodeling complex—MTA2, ACTL6A, CBX5, SMARCA4
Antigen processing and presentation of peptide antigen via MHC class I—H2-D1, FCER1G, B2M, TAPBP	
Apoptotic mitochondrial changes—SH3GLB1, BAX, SOD2	
Cellular lipid catabolic process—HEXA, PLCG2, HEXB, HADHA, ACOX3, HADHB	

### Disruptions in chromatin remodeling and transcriptional regulation

When examining functional categories including gene ontology functions and keywords, downregulated proteins were enriched for functions related to RNA processing and chromatin modification (Table 1). Terms like chromatin organization, helicase activity, regulation of transcription, and chromatin remodeling complex suggest aged microglia have impairments in their ability to appropriately regulate proteins at the epigenetic/transcriptional level. Central to the maintenance of the chromatin and epigenetic regulation are histone proteins. The histone linker protein Histone H1.5 (Hist1h1b) allows for the compaction of chromatin into higher order structures and is downregulated ~6-fold in our dataset (Fig. 1 and Additional files 1 and 2). Additionally, and related to their function in chromatin structure, histone proteins play a role in transcriptional regulation that is mediated through their post-translational modification code, including modifications like methylation, phosphorylation, and acetylation [49]. Proteins responsible for these modifications make up chromatin remodeling complexes of which NuRD and SWI/SNF are two examples [1]. Because proteins are enzymatically digested prior to identification, it is possible to detect a change in a particular segment of a protein that is unique to a specific isoform that may not reflect changes in overall protein level. It is possible that the reported downregulation could be an isoform-specific modification indirectly detected at the peptide level, which could implicate differential histone modifications that could occur with aging in microglia. This is an interesting area for future investigations. To further support the notion of diminished chromatin remodeling capability, core components of each (CHD4, MTA2) and (Smarca4,2, ACTL6a) are downregulated in our dataset (Additional file 2: Table S2). Both complexes are involved in the transcriptional regulation of macrophage polarization and inflammatory signaling. A prominent feature of aged microglia is the loss of polarization fluidity or the ability to switch from one activation state to another [37]. This switch requires several histone modifying complexes in order to adequately respond to environmental stimuli in a controlled fashion. Chromatin remodeling complexes are not only responsible for post-translation modifications of histones but also transcription machinery like the RNA polymerase. These modifications control the expression of early phase response genes like TNFα and IL1β during acute inflammatory reactions. Also, Toll-like receptor signaling (TLR)-induced histone acetylation is necessary for the induction of tolerance that occurs as a result of inflammatory gene activation [20]. The dynamic nature of histone modifications is central to the resolution of the inflammatory response and the protection of healthy brain tissue.

### Loss of nuclear architecture and impairments in RNA processing

Aged microglia also have substantial changes in the architecture of the nucleosome, an organelle whose proper function acts as a regulatory mechanism for transcription [39]. Table 1 shows a downregulation of a number of proteins involved in the structure of the nuclear lumen. The nuclear envelope is important not only for export and import of transcription factors and mRNA from the nucleus but also for the maintenance of higher-level chromatin structure [39]. This architecture, which includes both the nuclear lumen and chromatin, experiences degradation as a result of age, contributing to disruptions in cellular function [39]. This potential outcome is highlighted in Table 1 in which proteins involved in nucleocytoplasmic transport and chromatin organization are downregulated in aged microglia. Age-dependent changes in alternative splicing can affect the expression of decoy receptors for pro-inflammatory cytokines like TNFα and IL1β [34]. These receptors play an important role in the regulation of pro-inflammatory signaling. The pathway analysis in Table 1 shows a downregulation of the constituent proteins of the spliceosome complex. Nuclear import/export and changes in the proteasome may all combine to contribute to an increased number of dysfunctional or improperly translated proteins, an increase that can possibly trigger stress responses capable of inducing apoptosis and low-grade inflammation in immune cells. Upstream analysis in the next section identifies two proteins in particular XBP1 and SYVN1 which are indicative of activation of the ATF6 branch of the ER stress signaling pathway [12]. Occurring concurrently with the loss of nuclear architecture is a diminished DNA repair capacity. Priming of microglia has been observed in animal models deficient in DNA repair capability [42].The DNA repair KEGG pathway contains several downregulated proteins in Table 1. Breakdown of higher order chromatin structure and excessive DNA damage can both lead to the loss of transcriptional control necessary for the appropriate polarization of microglia in response to environmental stimuli.

### Aged microglia exhibit a bioenergetic shift from glucose to fatty acid utilization

A shift from the utilization of glucose to fatty acids and amino acids for energy production was first observed in the hippocampus of aged female mice [16]. The Brinton group postulated that this switch, to less efficient bioenergetic fuels, contributed to the increased oxidative environment of the aged brain. Table 1 highlights changes in mitochondrial function that suggest microglia, too, undergo this transition to alternate substrate utilization (Table 1). HADHA, HEXA, HEXB, and PLCG2 are all proteins whose upregulation is indicative of utilization of fatty acids and ketone bodies [52]. Bioenergetic stress, like that triggered by inflammation or nutrient deprivation, activate the protein nuclear respiratory factor 1 (NRF1); we observed a predicted activation of NRF1 in our dataset (Table  2 ). NRF1 leads to increases in the expression of components of the electron transport chain (ETC) and mitochondrial membrane; this can be seen in our analysis of upstream regulators (Table 2). Astrocytes in the hippocampus of male mice have shown an age-dependent increase in processes like lipid catabolism, proteolysis, and cholesterol transport [27]. One theory put forth by the Landfield group is that this metabolic change in astrocytes results in increased cholesterol trafficking in the local environment [27]. One molecular mechanism that governs the utilization of the most abundant nutrient sources, glucose and fatty acids, is called the “Randle cycle” and is thought to be important in insulin resistance, which increases with age [4]. This shift in substrate utilization decreases neuronal function and may precede cognitive dysfunction [27]. The consequences of this shift have not yet been studied in microglia but there is an established connection with this substrate shift and oxidative stress and inflammation. In addition to increased ROS, there is also evidence that myelin phagocytosis by microglia can impair their function and elicit a dystrophic phenotype reminiscent of aged cells [44]. The number of microglia is increased in the white matter of aged brains [45], and we have observed an increase in several myelin-related proteins. It is possible that microglia as well as astrocytes contribute to increased cholesterol trafficking that contributes to neuronal dysfunction with age. This possible mechanism is supported by the identification of APOE as an upregulated protein in aged microglia (Additional file 2: Table S2). Myelin-oligodendrocyte basic protein is the third most abundant myelin protein in the CNS and is also upregulated ~6-fold in this data set (Additional file 2: Table S2). One study demonstrated that even one demyelination event can increase insoluble lysosomal myelin aggregates in microglia and result in an upregulation of markers of activation like MHCII [44]. Unlike in astrocytes, a direct link between the Randle cycle shift and phagocytosis of myelin by microglia has not been identified. However, metabolic reprogramming is a central feature of macrophage and microglial polarization [21]. While the consequences of a Randle cycle shift in microglia are not fully elucidated, it is plausible that it would have a meaningful impact on cellular function similar to the one seen in neurons.

**Table 2:** Upstream regulators from IPA analysis

Top 10 upstream regulators by *p* value	*p* value of overlap	Activated upstream regulators by *z*-score	*z*-score	Inhibited upstream regulators by *z*-score	*z*-score
RICTOR	3.55E−12	INSR	3.43	MAP4K4	−3.00
MYC	3.21E−11	IFNG	3.37	RICTOR	−2.84
Nrf1	1.42E−10	IKBKB	3.14	VCAN	−2.00
NFE2L2	1.20E−09	APP	3.11		
CD3	1.22E−09	NRF1	2.97		
TP53	7.91E−09	TP53	2.58		
HNF4A	1.83E−08	XBP1	2.56		
INSR	3.49E−08	Ins1	2.53		
TGFB1	8.12E−08	CST5	2.5		
CST5	1.37E−07	Cdc42	2.45		

### Pathway clustering and upstream regulators

**Fig. 2:**
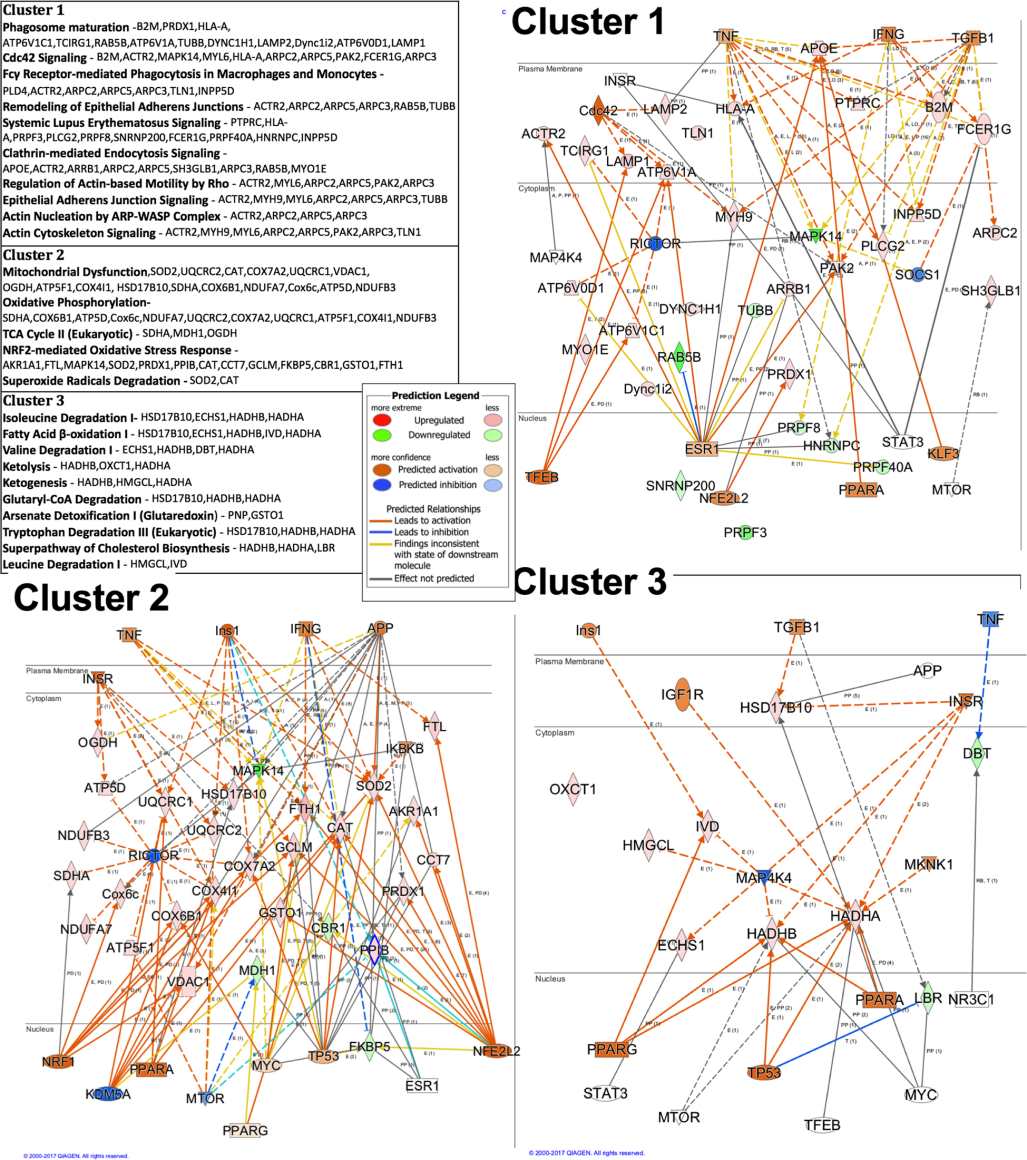
Canonical pathways form groups for each individual protein. The table on the *left* contains the name of each pathway in the cluster, as well as the proteins for which the pathway enriches. To illustrate the interrelationships of the proteins in each cluster with upstream regulators determined by IPA, the proteins that enriched for the pathways in clusters 1, 2, and 3 as well as upstream regulators were visualized as a network using IPA. Downstream proteins that were detected in the proteomics experiment are denoted by fold change and *p* values beneath them. *Red color* indicates an upregulation and *green* a downregulation with color intensity related to expression value. Upstream regulators are listed in the table to the *right* of the figure and contain the downstream proteins effected. Upstream regulator are colored *with orange* (upregulation) or *blue* (downregulation). The lines connecting various proteins are meant to describe the predicted effect that the observed change has on that particular relationship. A *blue line* would suggest that due to the differential expression of the upstream protein, it is having an inhibitory effect on the downstream target. With this graphic representation of the networks, the complex interactions and nodes of intersection can be presented. For example, note that RICTOR, one of the top upstream regulators identified, is central to both clusters 1 and 2

Ingenuity uses statistical methods to determine and score regulators whose network connections are unlikely to happen in a random model. The *p* value of overlap scores the enrichment of network-connected genes for a particular molecule. While the activation *z*-score identifies molecules using a statistically significant pattern match of up- and downregulation, the *z*-score also predicts the activation state of the putative regulator. The primary objective of upstream analysis is to identify molecules whose activity has broad effects on the cellular systems that are altered in the data set (Table 2). To that end, and to gain an understanding of the pathways that constituted the many functions discussed in the previous section, we first used Ingenuity to create a map of overlapping canonical pathways (Fig. 2). The top 25 canonical pathways significance of overlap were arranged according to shared proteins, an indicator of co-regulation. Figure 2 lists three clusters of pathways that are categorized according to function and downstream biology. Cluster 1 is predominated by pathways involved in phagocytosis and cytoskeletal reorganization. Cluster 2’s pathways include oxidative phosphorylation and the tricarboxylic acid (TCA) cycle, clearly dealing with mitochondrial function. Finally, the third cluster shows several catabolic pathways involved in the degradation of amino acids and the oxidation of fatty acids and ketones. These clusters clearly mirror the biology discussed in the previous section and give us a point of reference with which to begin identifying upstream regulators capable of influencing the biology of each cluster. To further illustrate this point, we used IPA to illustrate the interactions of the proteins identified with each of the clusters to top upstream regulators associated with the proteins in each cluster (Fig. 2). Predicted upstream regulators are in blue (predicted inhibition) or orange (predicted activation). Proteins detected in our dataset in each cluster leading to these predictions are illustrated in red (upregulated) or green (downregulated).

### Cluster 1

The highest proportions of proteins in cluster 1 are enriched for phagosome maturation and FCγ receptor-mediated phagocytosis (Fig. 2). The remaining pathways in the cluster involve actin polymerization and remodeling and signaling for auto-immune disease. The two principal mechanisms involved in phagocytosis are compliment receptor 3 (CR3) and FCγ [8]. CR3 can phagocytose complement opsonized or unopsonized targets while FCγ requires prior opsonization by antibodies [8]. FCγ-mediated phagocytosis is accompanied by pseudopod extension, respiratory burst, and production of cytokines including TNFα [8]. FCγ binding activates Cdc42/Rac signaling (identified as an upstream regulator in the analysis), which is responsible for the polymerization of actin in lamellipodia and fillopodia and is characteristic of FCγ-mediated phagocytosis. Activation of Cdc42/Rac leads to the upregulation of P38 MAP kinase, activation of NFκB, and subsequent upregulation of the NADPH oxidase enzyme complex responsible for the respiratory burst [51].

Upstream analysis of this cluster provided a list of potential regulators, the majority of which (five of eight) were related to cytokine signaling including TNF, IFNγ, and TGFβ1 (Fig. 2). The aged brain is characterized by higher levels of pro-inflammatory cytokines, making it plausible that increase phagocytosis is occurring as a result of an ongoing immune response [37]. Another important aspect of regulation of this cluster includes RICTOR and MYC that are regulators of proteins involved in formation of the lysosome. Both RICTOR and MYC interact with mTORC2, which is upstream of lysosomal proteins involved in the degradation of macromolecules [14]. Lysosomes play a role in cellular homeostasis and play a role in nutrient sensing through an interaction between TFEB and mTOR. The mTOR pathway is also involved in the innate immune response and can be activated by signals from several receptor types including TLRs and scavengers [53]. In the previous section, evidence for increased phagocytosis of myelin was presented. Increased expression of the MHCII receptor is greater in the white matter of the aged brain and may correlate with the enhanced degradation of myelin [37]. CR3 is considered to be the principle means by which myelin is phagocytosed by microglia; in addition, other receptors including FCγ have also been shown to be involved.

### Cluster 2

**Fig. 3:**
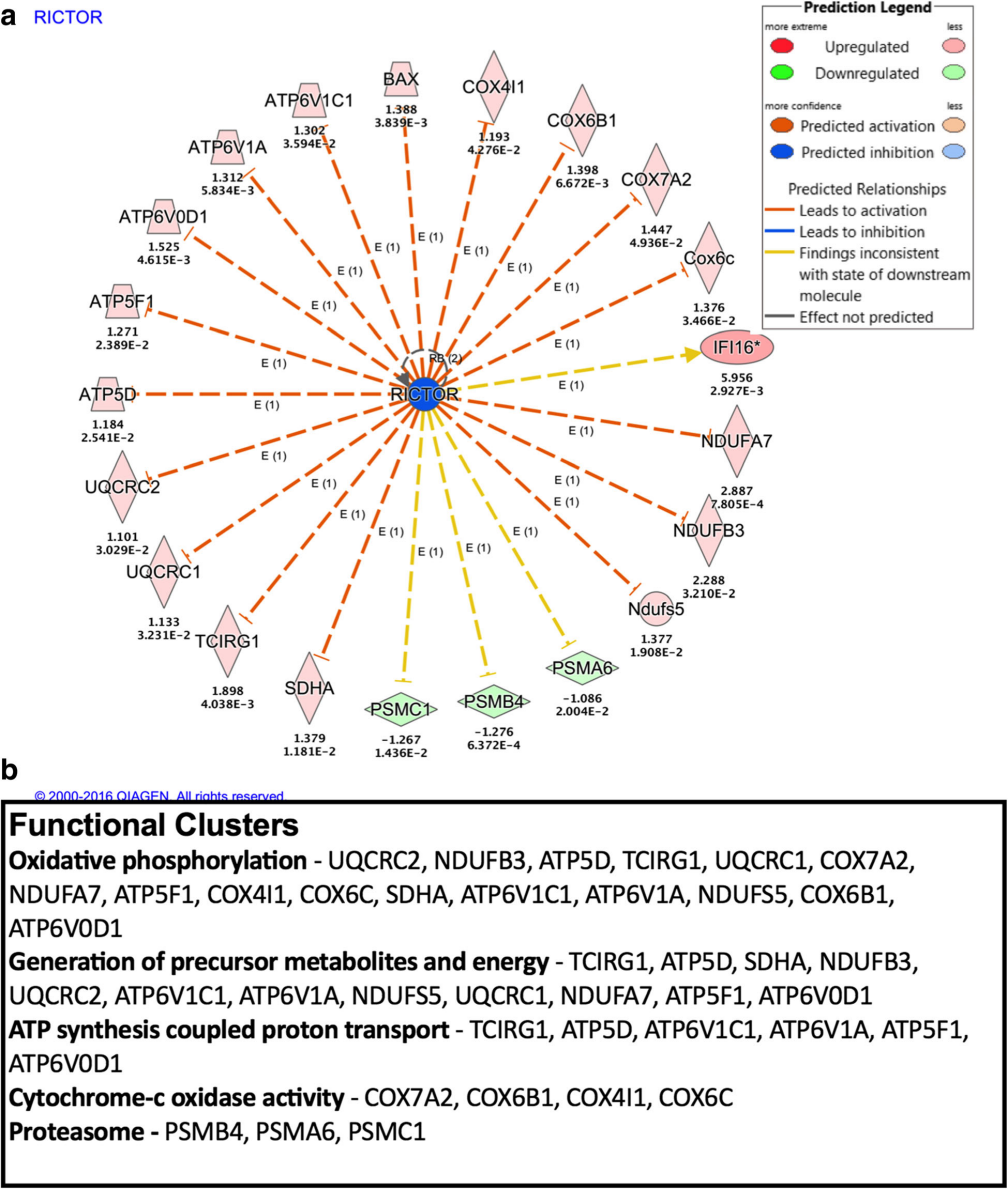
RICTOR is upstream of multiple canonical pathways. **a** RICTOR and its downstream proteins displayed as a network. *Symbol color* represents expression value, *red* indicating an upregulation and *blue*/*green* indicating downregulation in our dataset. RICTOR modulates the expression or function of downstream proteins listed in figure. The aggregate expression values of data set proteins contribute to a predicted decrease in RICTOR activity. **b** Proteins downstream of RICTOR can be grouped into five canonical pathway groups and four families. The identified pathways are also included in the top five pathways identified as affected by age

Cluster 2 contains pathways involved in mitochondrial function including oxidative phosphorylation, the TCA cycle, and NRF2-mediated oxidative stress response (Fig. 2). One upstream regulator NRF1 is responsible for the transcription of nuclear mitochondrial genes, including components of the electron transport chain. NRF1 is a downstream target of MYC but can also be activated through chronic activation of 5′-adenosine monophosphate-activated protein kinase (AMPK) due to bioenergetic stress, which is implicated in our dataset as well by predicted changes in insulin receptor signaling [3]. A protein of the TCA pathway, OGDH, is downstream of the insulin receptor and involved in the conversion of alpha-ketoglutarate to succincyl-CoA and is indicative of utilization of ketones in the Krebs cycle [16]. The increase in mitochondrial subunits via NRF1 may be related to a possible shift to ketones as an energy source. Ketones and fatty acids are less efficient than glucose as energetic substrates, producing more ROS per unit ATP. The Randle cycle, a shift to the most abundant substrate available, under normal circumstances can be overridden through the signaling of AMPK, which is activated during periods of nutrient scarcity. In fact, AMPK activity is upstream of NRF1 and activates during energetic stress [3]. It is possible that disruptions in parallel nutrient-sensing pathways like mTOR are interfering with the cells’ ability to utilize glucose. Nutrient-responsive pathways predominate the upstream regulators, with insulin, mTOR, RICTOR, and peroxisome proliferator-activated receptor alpha (PPARα) signaling all implicated in this cluster. NFE2L2 is also present as a predicted regulator, upstream of proteins involved in response to oxidative stress (Fig. 3). KDM5A also known as JARD1A is a histone 3 lysine 4 demethylase that binds to several nuclear transcription factors including c-MYC, PGC-1α, and the tumor suppressor protein pRB. Loss of KDM5A increases mitochondrial respiration as KDM5A is a direct repressor of metabolic regulator genes. Both nutrient-sensing and histone-modifying pathways are altered with age as reviewed [31]. Cluster 2 demonstrates direct links between these pathways and mitochondrial function. It is unclear how these mitochondrial changes contribute to the aged microglial phenotype. However, increased NRF2 signaling may be an indicator of increased mitochondrial ROS production, which is an indicator of mitochondrial dysfunction. Furthermore, mitochondria as the powerhouse of the cell can impact numerous cellular functions important for microglial function.

### Cluster 3

This cluster has a particular relevance to the aged phenotype. This group of pathways governs conversion of metabolic intermediates into substrates for oxidative phosphorylation (Fig. 2). Dominant here are proteins responsible for the breakdown of amino acids and the conversion of ketone bodies into succinyl-CoA. In addition to the utilization of amino acids, this cluster is enriched for proteins necessary for the oxidation of fatty acids. Upstream of these proteins are relevant nutrient-sensing pathways, mTOR, PPARα/γ, and the insulin receptor (Table 2). These pathways govern a large network of metabolic genes that control the shuttling and processing of macromolecules used in bioenergetic processes [17].The second group of upstream regulators are mediators of the inflammatory response and include TGFβ, p53, and MAP4K4. MAP4K4 facilitates the signal transduction of TNFα signaling while p53 is a transcriptional regulator involved in NFκB signaling and has been recently implicated as a regulator of the microglial phenotype [26]. Seeing the involvement of inflammatory mediators upstream of these pathways suggests that this shift in substrate utilization may be due to an ongoing inflammatory process. There is currently no evidence that acute activation of microglia triggers this shift, although metabolic reprogramming is a central component of innate immune cell polarization [28]. While TLR4 activation has been shown to shift innate immune cells towards reliance on glycolysis, this has not been observed by activators other than LPS [28]. In fact, this cluster is more consistent with alternative activation of macrophages. This finding is consistent with the idea that phagocytosis is a function of alternatively activated microglia but contrary to data suggesting aged microglia exist in a pro-inflammatory state with blunted anti-inflammatory responses. Results from these studies embody the inherent complexity of microglial polarization and underlie the difficulty in defining “activation states” of this cell type based on observations made in vitro*.*

The goal of this upstream analysis was to find proteins capable of affecting underlying biology through their interaction with the various affected cellular systems. RICTOR, the primary component of the mTORC2 complex, was the top scoring regulator with respect to both *z*-score and *p* value. In this analysis, RICTOR was directly implicated in two of the three clusters and, more broadly, mTOR signaling was predicted to regulate at least one pathway in each cluster. Thus, we examined a potential role for inhibition of mTORC2 in regulating microglial phenotype.

### mTORC2 as an upstream regulator of microglial phenotype

**Fig. 4:**
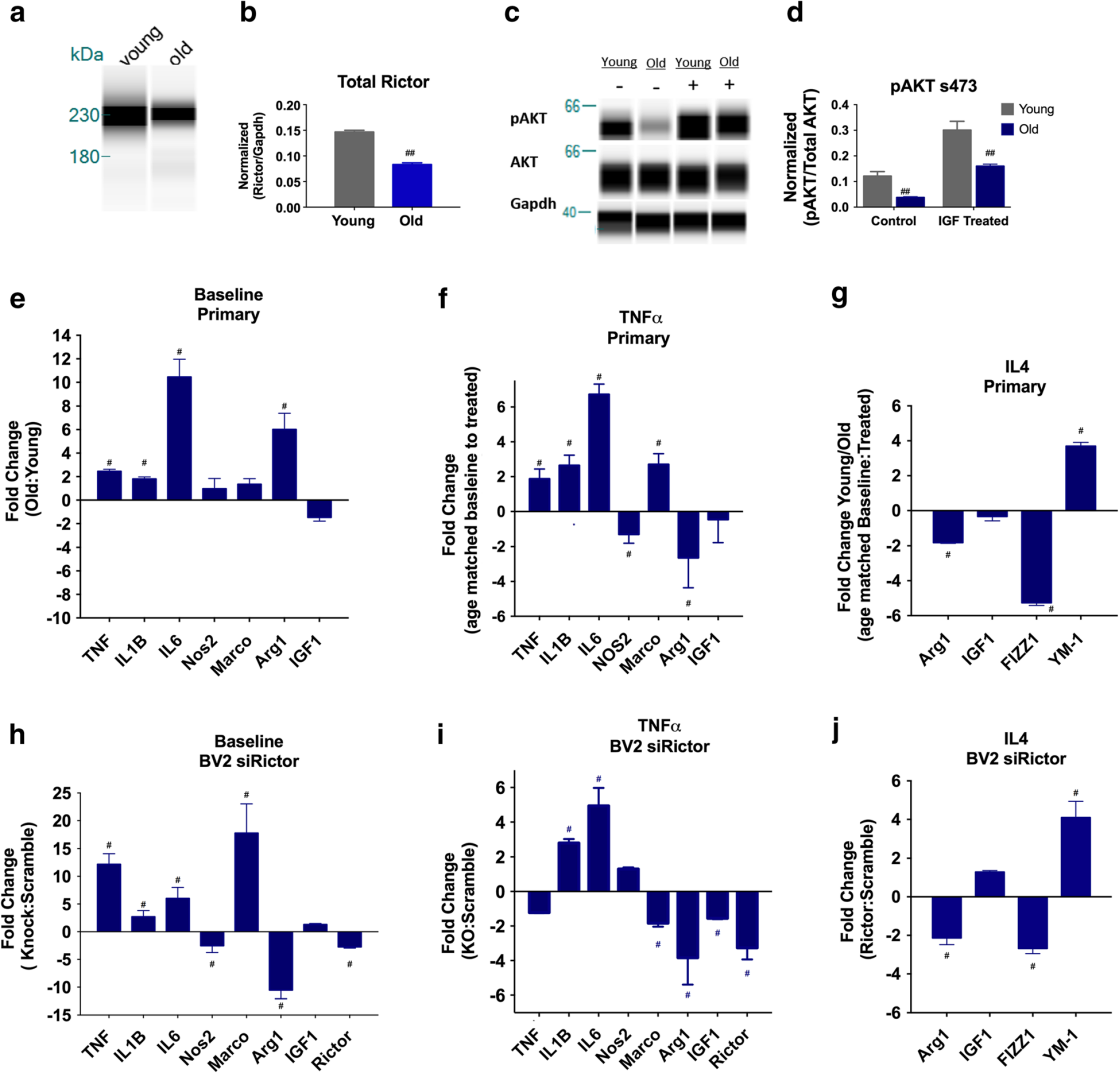
Age-dependent decrease in mTORC2 contributes to pro-inflammatory phenotype. **a** Protein analysis using the Wes instrument (Protein Simple) analyzing levels of RICTOR in primary microglia. **b** Graph representing measurements from RICTOR western show aged microglia have diminished levels of the RICTOR protein (^##^ *p* < 0.01 Student’s unpaired *t* test, *N* = 3). **c** Primary microglia young (5–7 months) and old (20–24 months) were treated with 100 ng/ml IGF for 15 min and then protein samples analyzed for AKT and AKTp473. **d** Analysis of intensity measurements received from the WES indicates pAKT activation by IGF is lower in aged microglia (two-way ANOVA, there was a significant effect of age *F*(1,8) = 60.8, ^##^ *p* < 0.01 and a significant effect of treatment *F*(1,8) 33.45 ^##^ *p* < 0.01. *N* = 3). **e** PCR analysis of primary microglia shows increased pro-inflammatory gene expression at baseline; values are expressed as fold change from young. **f** Primary microglia were stimulated with 20 ng/ml TNFα for 24 h. **g** Aged microglia have higher change from their respective baseline than young microglia for TNF, IL1β, IL6, and Marco. **h** Primary microglia were stimulated with IL4 20 ng/ml for 24 h. A reduced change (>−1.5-fold) from baseline in the aged compared to control was observed for Arg1 and FIZZ1. **i** BV2 cells (siRNA Rictor 48 h) treated with TNFα had higher levels of inflammatory gene expression IL1β and IL6 than a scramble control group. **j** BV2 cells pretreated with siRNA and scramble RNA for 48 h and subsequently treated with IL4 (20 ng/ml) for 24 h had lower expression of genes Arg1 and Fizz1 associated with resolution of the inflammatory response. Fold changes for baseline that are reported are relative comparisons using Young/Scramble as control. Interpreted as had lower expression of anti-inflammatory genes as compared to young microglia. *N* = 3 biological replicates per experiment examined in triplicate. For the TNFα and IL4 stimulation experiments, data is represented as the ratio of fold change from age/treatment matched control. Interpreted as the fold change from baseline in aged microglia for IL6 in response to TNFα is sevenfold higher than that observed in young microglia. Fold change of greater than 1.5 is considered significant (marked by *number sign*), all changes are illustrated, not just those that met criterion

Dysregulated polarization of microglia is considered a primary target for the treatment of cognitive dysfunction underlying both normal aging and neurodegenerative disease. This study has outlined potential proteomic changes related to age-dependent microglial dysfunction. The mTORC2 pathway was the top scoring upstream regulator with respect to both *z*-score and *p* value; in addition, it had the highest diversity of proteins and canonical pathways in the dataset that were responsible for the predicted downregulation of mTORC2 activity (Fig. 3). A literature review of mTOR’s role in inflammation underscores a role for mTORC2 in the regulation of innate immune [6, 7]. The mTOR pathway senses nutrient abundance and is a master regulator of cellular metabolism and growth [5]. mTOR exerts influence over innate immune cell activation through its coordination of the metabolic reprogramming that occurs during polarization [53]. Although the mechanism by which mTORC2 specifically exerts its effect on microglia is unknown, several studies offer potential downstream actions that may regulate inflammatory pathways. These include mTORC2’s action to phosphorylate AKT at serine 473, which is obligatory for its activity to regulate both FOXO1 and a network of miRNAs responsible for regulation of the pro-inflammatory response. To confirm that mTORC2 activity is diminished in aged microglia, we examined RICTOR protein expression and the ability of IGF to stimulate phosphorylation of AKT at s473 in young and aged primary microglia from C57BL/6N mice (Fig. 4a–d). We show a diminished level of RICTOR protein in the isolated primary microglia of aged mice (Fig. 4a, b). mTORC2 is regulated by nutrient-sensing pathways involved in energy regulation as both insulin and IGF have been shown to trigger mTORC2-dependent phosphorylation of AKT at serine 473 [25]. We examined this pathway in our isolated primary microglia, both at baseline and 15 min post-IGF exposure. Aged microglia had significantly lower phosphorylation AKT at s473, which is consistent with the prediction made by IPA (Fig. 4c, d). To determine whether diminished mTORC2 activity was sufficient to induce an inflammatory response similar to that of aged microglia, we used a siRNA-specific for RICTOR in BV2 cells and compared that with baseline, TNFα-, and IL4-stimulated response of primary microglia from young and aged mice. The profile of RICTOR knockdown cells shared significant overlap with aged primary cells, which, at baseline, have increased expression of TNFα, IL1β, and IL6 represented as fold change from young or the negative reciprocal (Fig. 4e, h). Further similarity is observed when cells were exposed to the pro-inflammatory cytokine TNFα. Both aged cells and BV2 cells with diminished mTORC2 activity had higher levels of IL1β and IL6 (Fig. 4f, i). It is worth noting that both after TNFα and IL4 treatment increased the expression of the gene Marco, once thought to be a primarily pro-inflammatory marker. Another feature of aged microglia is failure to upregulate anti-inflammatory molecules indicative of alternative activation states. Therefore, the response to IL4 was also examined. Aged microglia exhibit a decreased upregulation of both Arg1 and Fizz1 while showing no change in IGF and an increase in the gene YM-1 (Fig. 4g). Microglia with diminished mTORC2 activity exhibited a strikingly similar profile, where induction of both Arg1 and Fizz1 were blunted while expression of YM-1 was higher than cells with RNA-scramble treated control (Fig. 4j).

While these results are promising, we examined a significant but limited subset of genes that delineate between pro- and anti-inflammatory states of the cell [46]. Other factors such as phagocytic activity, ROS production, and neurotrophic factor production are features of microglial activation that affect the development of age-dependent disease presentation and progression [33]. Even with this caveat, the results are significant enough to warrant further exploration of the role of mTORC2 in the aged microglial phenotype and if this alteration is functionally involved in aspects of the loss of resilience of the aged brain to respond to injury and progression of neurodegenerative diseases.

## Conclusions

Primed microglia have been described in both aging and neurodegenerative disease [40]. They are characterized as having hypersensitive responses to pro-inflammatory stimuli, diminished responses to immuno-modulatory molecules, impaired phagocytic capability, and a dystrophic morphology [40]. It is believed that this contributes to the progression of neurodegenerative diseases including AD and Parkinson’s disease [22, 37]. Microglia are remarkably versatile cells exhibiting a wide range of activation states that have been characterized using the M1 classical and M2 alternative states [50]; however, it is widely recognized that this classification does not fully describe the pleiotropic nature of the response of microglia to the environment. This versatility is a necessary component for microglia to maintain a homeostatic and resilient environment through communication with their environment. In one sense, aging represents a loss of that resiliency characterized, in part, by the loss of transcriptional control over their responses and a reduced ability to sense the surrounding signals. The molecular mechanisms responsible for this age-specific phenotype, sometimes referred to as priming, remain unknown although the transcriptional profile of these cells is becoming more clear [24]. The transcriptome of aged microglia significantly overlaps with that of microglia found in degenerative disease but, to no surprise, bears little relationship to that of solely either classical or alternatively activated [38]. When interpreting our results, it was important to consider the possible inclusion of perivascular macrophages, which may have been isolated along with microglia. Because CD11B is present on both cell types, it is possible that these cells were present throughout the analysis. We took several steps to mitigate this potential shortcoming, including removal of meninges before isolation and the use of the BV2 cell line as our source of heavy-labeled peptides during mass spectrometry. Microglia and macrophages have unique expression profiles [23], and significant macrophage infiltration would have resulted in a high number of unpaired light peptide chains that were specific to macrophages. While, it is currently not possible to ensure a 100% pure population of microglia, it is possible to mitigate this risk during processing.

One goal of this study was to better define the molecular pathways that contribute to the aged phenotype. Aged microglia have a marked decline in cellular systems responsible for transcriptional control. These include the nucleosome, spliceosome, and complexes responsible for the maintenance and modification of chromatin (Table 2). Induction of tolerance to inflammatory stimuli is one of the earliest events in inflammatory signaling and is dependent on modification of histones to restrict transcription factors that drive the response. In addition to a loss of regulatory control, aged microglia exhibit impaired DNA repair capability (Table 2). Models of priming in DNA repair-deficient microglia have significant phenotypic overlap with that of aged [42]. Whether or not these changes result directly in the hypersensitivity observed with age, they likely contribute to other maladaptive changes observed in microglia. A central component of the microglia shift in phenotype involves the reprogramming of metabolic networks that involves the mTOR pathway. The data presented in this study suggest aged microglia have shifted from the use of glucose to the oxidation of ketones and fatty acids for energy. While this has been observed in other cell types in the aged brain, this is the first time it has been identified in a purified microglial. Long-term use of these substrates can lead to increased mitochondrial ROS and may be indicative of loss of insulin [27]. Subsequently, this study has identified changes in two cellular systems that have the ability to directly impact the phenotype of aged microglia. What influence the environment plays on the age-related changes in microglia is currently unknown. It is theorized that ongoing, low-grade inflammation contributes to the priming of microglia [40]. The upregulated functions in this analysis suggest microglia are actively migrating and phagocytosing substances in their environment. Microglial density is higher in the white matter of aged brains, and myelin degradation positively correlates with both age and markers of [32]. Recent studies suggest that the degradative capacity of microglia for myelin is reached in mid-life. Aged microglia are immuno-positive for non-soluble aggregates of myelin in lysosomes [44]. In this example at least, the ongoing pathology of myelin degradation in the environment is capable of driving transcriptomic changes that resembled that of aged cells, although the question remains if dysfunction of the microglia itself is the primary alteration responsible for the degradation of the myelin or a response to ongoing pathology.

As chronic inflammation is associated with the degenerative disease process, it is also a target for therapeutic intervention. The identification of RICTOR as an upstream regulator of several affected systems presented an opportunity to study both of the role of this pathway in microglial aging and its potential as a therapeutic target. Diminished mTORC2 activity affects several cellular systems. While this presents a challenge for defining a mechanism of action, it presents an opportunity for further understanding of the aged phenotype. It is likely that all of the pathways discussed in this section contributes a portion to the overall phenotype of the aged cell. However, the knockdown of the mTORC2 was able to recapitulate the majority of the phenotype measured in this report. This could result from other upstream changes that converge at mTORC2, or that several alterations downstream of mTORC2 within its regulatory network may be responsible for the effect observed when RICTOR is inhibited. In any case, this study has thoroughly examined the phenotype of the aged microglia at the proteome level and proposed several new theories regarding the priming of these cells. Future work will continue the validation of other regulators in this study to better understand how the complex signaling networks interact in the aged environments.

## Additional files

Additional file 1: **Table S1.** Differentially expressed proteins used for bioinformatic analysis (Student’s *t* test only) (PDF 66 kb)

Additional file 2: **Table S2.** Differentially expressed proteins filtered by Welch’s *t* test (*p* < 0.05) and |*z*-score| >1. (XLSX 19 kb)

## Abbreviations

AMPK: 5′-Adenosine monophosphate-activated protein kinase
CNS: Central nervous system
CR3: Compliment receptor 3
DNA: Deoxyribonucleic acid
IL: Interleukin
M1: Microglia or macrophage 1
M2: Microglia or macrophage 2
MHC: Major histocompatibility complex
mTOR: Molecular target of rapamycin
Nrf1: Nuclear respiratory factor 1
RICTOR: Rapamycin-insensitive companion of mTOR
RNA: Ribonucleic acid
ROS: Reactive oxygen species
SILAC: Stable isotope labeling of amino acids
siRNA: Short inhibiting ribonucleic acid
TCA: Tricarboxylic acid
TGFβ: Transforming growth factor β
TLR: Toll-like receptor
TNF: Tumor necrosis factor
