# Supplementary material for: Proteomic anaysis of aged microglia: shifts in transcription, bioenergetics, and nutrient response
Source: J Neuroinflammation. 2017 May 3;14:96. doi: 10.1186/s12974-017-0840-7 (PMC5415769; doi:10.1186/s12974-017-0840-7)
Supplement: Supplementary file 1 — Table S1. Differentially expressed proteins used for bioinformatic analysis (Student’s t test only) (PDF 66 kb) [file 12974_2017_840_MOESM1_ESM.pdf]

Exp Fold Change	Exp p-value	Protein/ Gene Symbol	Entrez Gene Name
21.553	2.15E-03	CYBA	cytochrome b-245, alpha polypeptide
16.640	2.78E-06	VPS52	VPS52 GARP complex subunit
10.753	1.97E-02	RAB8B	RAB8B, member RAS oncogene family
10.022	2.43E-02	SFN	stratifin
8.433	8.85E-04	PRKAR1A	protein kinase, cAMP-dependent, regulatory subunit type I alpha
6.800	2.41E-04	MOBP	myelin-associated oligodendrocyte basic protein
5.956	2.93E-03	IFI16	interferon, gamma-inducible protein 16
5.040	7.65E-04	FTH1	ferritin, heavy polypeptide 1
4.621	9.74E-03	PLEC	plectin
4.238	1.69E-03	APOOL	apolipoprotein O-like
4.108	2.49E-03	TOR3A	torsin family 3, member A
4.107	4.80E-02	LAMP1	lysosomal associated membrane protein 1
4.061	3.73E-02	SRP19	signal recognition particle 19kDa
3.938	7.52E-03	OAS1	2'-5'-oligoadenylate synthetase 1
3.687	1.26E-02	DHRS1	dehydrogenase/reductase (SDR family) member 1
3.574	4.22E-02	LGALS3BP	lectin, galactoside-binding, soluble, 3 binding protein
3.530	3.35E-02	NT5C2	5'-nucleotidase, cytosolic II
3.357	3.69E-02	LY86	lymphocyte antigen 86
3.298	1.26E-02	FTL	ferritin, light polypeptide
3.072	1.49E-02	NOMO1 (incl	NODAL modulator 1
3.051	1.69E-03	ASL	argininosuccinate lyase
3.021	3.39E-02	MTX2	metaxin 2
2.997	3.71E-03	USP48	ubiquitin specific peptidase 48
2.987	4.99E-02	SYNGR1	synaptogyrin 1
2.951	2.30E-02	GCLM	glutamate-cysteine ligase, modifier subunit
2.887	7.80E-04	NDUFA7	NADH:ubiquinone oxidoreductase subunit A7
2.834	3.43E-02	ADSSL1	adenylosuccinate synthase like 1
2.795	4.75E-02	ME2	malic enzyme 2, NAD(+)-dependent, mitochondrial
2.793	2.43E-06	HEXB	hexosaminidase B (beta polypeptide)
2.675	2.64E-02	CBR4	carbonyl reductase 4
2.547	2.37E-03	LSP1	lymphocyte-specific protein 1
2.529	7.29E-05	HEXA	hexosaminidase A (alpha polypeptide)
2.500	1.92E-03	SCARB2	scavenger receptor class B member 2
2.461	1.35E-02	H1F0	H1 histone family member 0
2.400	8.96E-03	RAB32	RAB32, member RAS oncogene family
2.388	1.46E-02	NRDC	nardilysin convertase
2.386	1.06E-02	MTX1	metaxin 1
2.363	3.89E-02	ZCCHC8	zinc finger, CCHC domain containing 8
2.349	3.29E-02	HINT1	histidine triad nucleotide binding protein 1
2.299	1.52E-02	ARRB1	arrestin, beta 1
2.288	3.21E-02	NDUFB3	NADH:ubiquinone oxidoreductase subunit B3
2.262	3.49E-02	ACOT9	acyl-CoA thioesterase 9
2.227	3.18E-02	CREB1	cAMP responsive element binding protein 1
2.169	3.75E-02	B2M	beta-2-microglobulin
2.164	2.62E-02	ACP2	acid phosphatase 2, lysosomal
2.156	4.51E-02	APOE	apolipoprotein E
2.070	2.83E-03	CTSZ	cathepsin Z
2.054	2.26E-02	Rpl36a	ribosomal protein L36A

2.050	2.98E-02	CD47	CD47 molecule
2.006	1.75E-02	ARL8A	ADP ribosylation factor like GTPase 8A
2.004	3.42E-02	ATPIF1	ATPase inhibitory factor 1
2.003	3.20E-03	GLTP	glycolipid transfer protein
1.917	7.86E-03	CORO1C	coronin 1C
1.898	4.04E-03	TCIRG1	T-cell, immune regulator 1, ATPase, H+ transporting, lysosomal V0 subunit A3
1.893	3.10E-02	TMA7	translation machinery associated 7 homolog
1.883	4.03E-02	WDR5	WD repeat domain 5
1.870	1.41E-02	CYB5R1	cytochrome b5 reductase 1
1.862	9.95E-03	IFI16	interferon, gamma-inducible protein 16
1.840	2.18E-02	HLA-A	major histocompatibility complex, class I, A
1.834	7.92E-03	SH3GLB1	SH3-domain GRB2-like endophilin B1
1.821	3.33E-02	FCER1G	Fc fragment of IgE receptor Ig
1.817	3.84E-02	TIAL1	TIA1 cytotoxic granule-associated RNA binding protein-like 1
1.765	1.69E-02	Dync1i2	dynein cytoplasmic 1 intermediate chain 2
1.757	7.79E-04	ECHS1	enoyl-CoA hydratase, short chain, 1, mitochondrial
1.713	8.59E-04	PHB	prohibitin
1.710	4.94E-02	GPS1	G protein pathway suppressor 1
1.687	2.63E-03	ANXA5	annexin A5
1.678	9.14E-03	CAPG	capping protein (actin filament), gelsolin-like
1.672	6.31E-05	SAR1A	secretion associated, Ras related GTPase 1A
1.664	2.26E-02	MYO1E	myosin IE
1.640	2.27E-04	GSTO1	glutathione S-transferase omega 1
1.637	4.05E-02	TRA2A	transformer 2 alpha homolog (Drosophila)
1.608	1.04E-03	RPS14	ribosomal protein S14
1.601	1.53E-03	LAMP2	lysosomal associated membrane protein 2
1.564	2.52E-03	INPP5D	inositol polyphosphate-5-phosphatase D
1.554	2.46E-02	HSD17B11	hydroxysteroid (17-beta) dehydrogenase 11
1.549	3.45E-03	PHB2	prohibitin 2
1.547	1.36E-03	NPC1	Niemann-Pick disease, type C1
1.529	6.97E-03	AIF1	allograft inflammatory factor 1
1.525	4.62E-03	ATP6V0D1	ATPase, H+ transporting, lysosomal 38kDa, V0 subunit d1
1.525	2.77E-02	SPCS3	signal peptidase complex subunit 3
1.521	1.66E-03	ACOT13	acyl-CoA thioesterase 13
1.512	2.67E-02	TAPBP	TAP binding protein (tapasin)
1.508	2.28E-02	ACOX3	acyl-CoA oxidase 3, pristanoyl
1.505	3.49E-05	GATM	glycine amidinotransferase
1.504	1.66E-03	ARL8B	ADP ribosylation factor like GTPase 8B
1.481	7.06E-04	HK2	hexokinase 2
1.479	9.48E-04	STX7	syntaxin 7
1.473	2.20E-03	LGALS9B	lectin, galactoside-binding, soluble, 9B
1.468	1.76E-02	ATL3	atlastin GTPase 3
1.462	3.69E-03	HMGCL	3-hydroxymethyl-3-methylglutaryl-CoA lyase
1.457	2.89E-02	CD180	CD180 molecule
1.455	5.36E-03	ACADS	acyl-CoA dehydrogenase, C-2 to C-3 short chain
1.452	5.62E-03	PTPRC	protein tyrosine phosphatase, receptor type C
1.448	2.03E-02	APOA1BP	apolipoprotein A-I binding protein
1.447	4.94E-02	COX7A2	cytochrome c oxidase subunit VIIa polypeptide 2 (liver)
1.443	3.45E-02	MYO1F	myosin IF

1.437	1.90E-03	HADHB	hydroxyacyl-CoA dehydrogenase/3-ketoacyl-CoA thiolase/enoyl-CoA hydratase (trifunctional protein), beta subunit
1.430	2.54E-02	ANXA4	annexin A4
1.429	1.99E-03	SRI	sorcin
1.404	5.58E-03	VAMP8	vesicle associated membrane protein 8
1.399	1.91E-02	OXCT1	3-oxoacid CoA-transferase 1
1.398	6.67E-03	COX6B1	cytochrome c oxidase subunit VIb polypeptide 1 (ubiquitous)
1.394	2.85E-02	SHMT2	serine hydroxymethyltransferase 2 (mitochondrial)
1.392	7.56E-03	RBM3	RNA binding motif (RNP1, RRM) protein 3
1.389	1.94E-03	NUP85	nucleoporin 85kDa
1.388	3.84E-03	BAX	BCL2-associated X protein
1.382	9.21E-04	PLCG2	phospholipase C gamma 2
1.379	2.03E-02	ACTR2	ARP2 actin-related protein 2 homolog (yeast)
1.379	1.18E-02	SDHA	succinate dehydrogenase complex subunit A, flavoprotein (Fp)
1.378	1.02E-03	PGD	phosphogluconate dehydrogenase
1.378	3.08E-02	BLVRB	biliverdin reductase B
1.377	1.91E-02	Ndufs5	NADH dehydrogenase (ubiquinone) Fe-S protein 5
1.376	3.47E-02	Cox6c	cytochrome c oxidase subunit VIc
1.375	3.20E-03	EIF3G	eukaryotic translation initiation factor 3 subunit G
1.369	9.18E-03	TECR	trans-2,3-enoyl-CoA reductase
1.369	1.76E-03	MTCH2	mitochondrial carrier 2
1.355	4.17E-02	IVD	isovaleryl-CoA dehydrogenase
1.354	3.77E-02	HMHA1	histocompatibility (minor) HA-1
1.349	2.03E-02	NAMPT	nicotinamide phosphoribosyltransferase
1.349	1.19E-02	COTL1	coactosin-like F-actin binding protein 1
1.344	8.24E-05	ARPC2	actin related protein 2/3 complex subunit 2
1.338	9.25E-03	PAK2	p21 protein (Cdc42/Rac)-activated kinase 2
1.337	8.78E-05	GANAB	glucosidase, alpha; neutral AB
1.326	2.79E-02	GOT1	glutamic-oxaloacetic transaminase 1, soluble
1.323	7.46E-03	EIF3D	eukaryotic translation initiation factor 3 subunit D
1.321	2.87E-02	ARPC5	actin related protein 2/3 complex subunit 5
1.317	4.39E-02	RPS6KA1	ribosomal protein S6 kinase, 90kDa, polypeptide 1
1.312	5.83E-03	ATP6V1A	ATPase, H+ transporting, lysosomal 70kDa, V1 subunit A
1.310	5.77E-04	SLC25A11	solute carrier family 25 (mitochondrial carrier; oxoglutarate carrier), member 11
1.302	3.59E-02	ATP6V1C1	ATPase, H+ transporting, lysosomal 42kDa, V1 subunit C1
1.301	6.73E-03	PLD4	phospholipase D family member 4
1.297	2.52E-02	DBNL	drebrin-like
1.291	7.25E-03	RAB2A	RAB2A, member RAS oncogene family
1.286	2.94E-03	TLN1	talin 1
1.285	1.06E-03	GLUD1	glutamate dehydrogenase 1
1.281	3.26E-02	CCT7	chaperonin containing TCP1 subunit 7
1.281	2.42E-02	AKR1A1	aldo-keto reductase family 1, member A1 (aldehyde reductase)
1.277	2.59E-02	LIPA	lipase A, lysosomal acid, cholesterol esterase
1.277	3.56E-02	CTSB	cathepsin B

1.275	9.09E-03	PRDX1	peroxiredoxin 1
1.274	1.85E-03	UAP1L1	UDP-N-acetylglucosamine pyrophosphorylase 1 like 1
1.273	4.58E-04	CAPZB	capping protein (actin filament) muscle Z-line, beta
1.271	2.39E-02	ATP5F1	ATP synthase, H+ transporting, mitochondrial Fo complex subunit B1
1.268	8.27E-03	HADHA	hydroxyacyl-CoA dehydrogenase/3-ketoacyl-CoA thiolase/enoyl-CoA hydratase (trifunctional protein), alpha subunit
1.264	1.35E-03	PAFAH1B1	platelet activating factor acetylhydrolase 1b regulatory subunit 1
1.262	5.09E-04	DYNC1H1	dynein, cytoplasmic 1, heavy chain 1
1.261	4.97E-02	CAT	catalase
1.260	2.90E-02	OGDH	oxoglutarate (alpha-ketoglutarate) dehydrogenase (lipoamide)
1.254	3.62E-02	ARL6IP5	ADP ribosylation factor like GTPase 6 interacting protein 5
1.253	3.48E-02	MYL6	myosin light chain 6
1.253	5.00E-02	YWHAH	tyrosine 3-monooxygenase/tryptophan 5-monooxygenase activation protein, eta
1.252	2.31E-02	CORO1A	coronin 1A
1.250	1.82E-02	VARS	valyl-tRNA synthetase
1.249	1.62E-02	VPS35	VPS35 retromer complex component
1.246	7.61E-04	MYH9	myosin, heavy chain 9, non-muscle
1.234	3.29E-02	SOD2	superoxide dismutase 2, mitochondrial
1.227	4.40E-02	ACTR1A	ARP1 actin-related protein 1 homolog A, centractin alpha (yeast)
1.225	1.13E-02	VWA5A	von Willebrand factor A domain containing 5A
1.215	2.04E-02	PITPNA	phosphatidylinositol transfer protein alpha
1.210	4.15E-02	ANXA6	annexin A6
1.210	1.58E-02	PPIB	peptidylprolyl isomerase B
1.209	3.19E-02	LCP1	lymphocyte cytosolic protein 1 (L-plastin)
1.198	4.59E-02	PA2G4	proliferation-associated 2G4
1.195	1.80E-02	EIF3E	eukaryotic translation initiation factor 3 subunit E
1.193	4.28E-02	COX4I1	cytochrome c oxidase subunit IV isoform 1
1.186	4.47E-02	HSD17B10	hydroxysteroid (17-beta) dehydrogenase 10
1.186	4.39E-02	ARPC3	actin related protein 2/3 complex subunit 3
1.184	2.54E-02	ATP5D	ATP synthase, H+ transporting, mitochondrial F1 complex, delta subunit
1.179	2.21E-02	VDAC1	voltage dependent anion channel 1
1.176	1.56E-02	CPNE3	copine III
1.172	4.73E-02	TAGLN2	transgelin 2
1.171	2.70E-02	Rrbp1	ribosome binding protein 1
1.157	2.06E-02	CAP1	CAP, adenylate cyclase-associated protein 1 (yeast)
1.135	3.90E-02	DDOST	dolichyl-diphosphooligosaccharide--protein glycosyltransferase subunit (non-catalytic)
1.133	3.23E-02	UQCRC1	ubiquinol-cytochrome c reductase core protein I
1.101	3.03E-02	UQCRC2	ubiquinol-cytochrome c reductase core protein II
1.097	2.73E-02	RPN2	ribophorin II
-1.086	2.00E-02	PSMA6	proteasome subunit alpha 6
-1.150	3.60E-02	SNX12	sorting nexin 12

-1.177	2.68E-02	NONO	non-POU domain containing, octamer-binding
-1.213	3.16E-02	NUTF2	nuclear transport factor 2
-1.218	4.69E-02	DDB1	damage-specific DNA binding protein 1
-1.223	8.14E-03	Anp32b	acidic (leucine-rich) nuclear phosphoprotein 32 family, member B
-1.226	2.23E-02	SF3B2	splicing factor 3b subunit 2
-1.226	3.30E-02	RBM14	RNA binding motif protein 14
-1.228	2.24E-02	ELAVL1	ELAV like RNA binding protein 1
-1.232	2.80E-03	TRA2B	transformer 2 beta homolog (Drosophila)
-1.234	1.05E-02	HNRNPU	heterogeneous nuclear ribonucleoprotein U (scaffold attachment factor A)
-1.235	1.70E-02	SNRNP200	small nuclear ribonucleoprotein, U5 200kDa subunit
-1.237	1.60E-02	DDX5	DEAD-box helicase 5
-1.238	1.79E-02	DYNLL2	dynein, light chain, LC8-type 2
-1.238	2.08E-02	TARDBP	TAR DNA binding protein
-1.239	4.72E-02	U2AF2	U2 small nuclear RNA auxiliary factor 2
-1.251	2.66E-03	PRPF8	pre-mRNA processing factor 8
-1.253	4.11E-02	SON	SON DNA binding protein
-1.254	4.57E-02	HNRNPL	heterogeneous nuclear ribonucleoprotein L
-1.255	3.81E-02	ACTL6A	actin like 6A
-1.259	1.25E-02	PNP	purine nucleoside phosphorylase
-1.261	2.54E-02	SMC1A	structural maintenance of chromosomes 1A
-1.262	2.45E-04	Srsf5	serine/arginine-rich splicing factor 5
-1.263	1.19E-02	LMNB1	lamin B1
-1.267	1.44E-02	PSMC1	proteasome 26S subunit, ATPase 1
-1.268	5.98E-03	DDX17	DEAD-box helicase 17
-1.276	6.37E-04	PSMB4	proteasome subunit beta 4
-1.282	4.93E-02	HNRNPUL1	heterogeneous nuclear ribonucleoprotein U-like 1
-1.282	2.19E-03	CHD4	chromodomain helicase DNA binding protein 4
-1.289	3.33E-02	TCERG1	transcription elongation regulator 1
-1.307	6.30E-03	Rbmxl1	RNA binding motif protein, X-linked like-1
-1.312	8.88E-04	SMU1	smu-1 suppressor of mec-8 and unc-52 homolog (C. elegans)
-1.318	4.95E-02	HNRNPC	heterogeneous nuclear ribonucleoprotein C (C1/C2)
-1.325	8.97E-03	KPNA3	karyopherin alpha 3 (importin alpha 4)
-1.339	2.95E-02	FKBP5	FK506 binding protein 5
-1.343	7.36E-03	LBR	lamin B receptor
-1.355	4.57E-02	HCFC1	host cell factor C1
-1.356	4.47E-02	CBR1	carbonyl reductase 1
-1.357	4.80E-02	NCBP1	nuclear cap binding protein subunit 1
-1.363	1.04E-02	APEX1	APEX nuclease (multifunctional DNA repair enzyme) 1
-1.365	3.66E-03	FUBP3	far upstream element (FUSE) binding protein 3
-1.381	2.50E-02	GLO1	glyoxalase I
-1.385	6.07E-03	TUBB	tubulin beta class I
-1.400	1.56E-03	HNRNPLL	heterogeneous nuclear ribonucleoprotein L like
-1.404	1.74E-02	MTA2	metastasis associated 1 family member 2
-1.405	1.33E-02	ILF2	interleukin enhancer binding factor 2
-1.405	2.76E-03	MDH1	malate dehydrogenase 1

-1.419	6.97E-05	Anp32a	acidic (leucine-rich) nuclear phosphoprotein 32 family, member A
-1.445	5.13E-03	TMEM173	transmembrane protein 173
-1.450	4.62E-03	HNRNPH2	heterogeneous nuclear ribonucleoprotein H2 (H')
-1.462	5.56E-04	RUVBL1	RuvB-like AAA ATPase 1
-1.467	1.89E-02	PRPF40A	pre-mRNA processing factor 40 homolog A
-1.476	1.67E-02	SET	SET nuclear proto-oncogene
-1.493	2.65E-02	SUB1	SUB1 homolog, transcriptional regulator
-1.494	6.68E-03	HNRNPH1	heterogeneous nuclear ribonucleoprotein H1 (H)
-1.589	5.50E-03	SMARCC2	SWI/SNF related, matrix associated, actin dependent regulator of chromatin, subfamily c, member 2
-1.599	4.06E-02	ATP1A1	ATPase, Na+/K+ transporting, alpha 1 polypeptide
-1.632	2.73E-03	HNRNPR	heterogeneous nuclear ribonucleoprotein R
-1.657	1.48E-02	KPNA6	karyopherin alpha 6 (importin alpha 7)
-1.772	6.44E-03	DBT	dihydrolipoamide branched chain transacylase E2
-1.792	1.52E-02	GPD1L	glycerol-3-phosphate dehydrogenase 1-like
-1.853	3.16E-04	DCPS	decapping enzyme, scavenger
-1.877	8.13E-03	SEC13	SEC13 homolog, nuclear pore and COPII coat complex component
-1.905	6.61E-04	CTSC	cathepsin C
-2.328	4.09E-02	CRYZ	crystallin zeta
-2.608	4.04E-02	THYN1	thymocyte nuclear protein 1
-2.758	2.33E-02	STK24	serine/threonine kinase 24
-2.783	3.42E-02	NUP153	nucleoporin 153kDa
-2.887	3.15E-02	AGPS	alkylglycerone phosphate synthase
-2.906	3.92E-02	P2RY12	purinergic receptor P2Y, G-protein coupled, 12
-3.078	4.70E-03	CUL5	cullin 5
-3.084	4.12E-03	HNRNPH3	heterogeneous nuclear ribonucleoprotein H3 (2H9)
-3.132	1.10E-02	RRS1	ribosome biogenesis regulator homolog
-3.167	2.78E-02	Snrpg	small nuclear ribonucleoprotein polypeptide G
-3.307	8.51E-03	PRPF3	pre-mRNA processing factor 3
-3.721	1.39E-02	CBX5	chromobox 5
-3.802	3.99E-02	CSTF2	cleavage stimulation factor, 3' pre-RNA, subunit 2
-3.942	9.18E-03	SMARCD2	SWI/SNF related, matrix associated, actin dependent regulator of chromatin, subfamily d, member 2
-4.205	3.23E-02	TERF2IP	telomeric repeat binding factor 2, interacting protein
-4.292	3.70E-02	HSPH1	heat shock protein family H (Hsp110) member 1
-4.359	2.64E-02	RAB5B	RAB5B, member RAS oncogene family
-4.438	4.51E-02	FGD2	FYVE, RhoGEF and PH domain containing 2
-4.449	1.85E-02	SMARCA4	SWI/SNF related, matrix associated, actin dependent regulator of chromatin, subfamily a, member 4
-4.699	1.15E-02	CRYBA4	crystallin beta A4
-5.429	2.34E-02	IPO9	importin 9

-5.815	4.64E-02	MAPK14	mitogen-activated protein kinase 14
-5.991	2.88E-05	Hist1h1b	histone cluster 1, H1b
-11.682	5.86E-05	FRG1	FSHD region gene 1
